# Predicted Spatial Patterns of Suitable Habitats for *Troides aeacus* Under Different Climate Scenarios

**DOI:** 10.3390/insects15110901

**Published:** 2024-11-18

**Authors:** Biyu Liu, Xinqi Deng, Zhiqian Liu, Xinju Wei, Honghua Zhang, Danping Xu, Zhihang Zhuo

**Affiliations:** College of Life Science, China West Normal University, Nanchong 637002, China; biyuliuql@foxmail.com (B.L.); deng.xinqi@foxmail.com (X.D.); qnhtvxhp319123@foxmail.com (Z.L.); weixinjuxx@foxmail.com (X.W.); honghua_zhang@foxmail.com (H.Z.)

**Keywords:** *Troides aeacus*, climatic conditions, environmental variables, MaxEnt, potential distribution area centroid

## Abstract

*Troides aeacus* is the largest butterfly in China and is highly valued for its ornamental beauty. Due to *T. aeacus* being classified as a national second-class protected species in China, studying its spatial distribution is crucial for developing effective conservation measures. In this study, a total of 490 distribution points were obtained, and the potential distribution areas of the golden-sheathed *T. aeacus* were analyzed by using the maximum entropy model (MaxEnt) based on three different greenhouse gas emission scenarios, namely, SSP1-2.6, SSP2-4.5, and SSP5-8.5, in combination with nine important environmental variables. The results indicate that temperature and precipitation are the primary environmental factors influencing the suitable habitat of *T. aeacus*, with key variables including the minimum temperature of the coldest month (bio6), temperature annual range (bio7), mean temperature of the warmest quarter (bio10), annual precipitation (bio12), precipitation of the coldest quarter (bio19), and slope. The height distribution of *T. aeacus* in my country is in the area south of the Huaihe River in the Qinling Mountains, with a total area of 270.96 × 104 km^2^, accounting for 28.23% of the total area of China. According to future climate change conditions, as climate warming progresses, both low- and high-suitability areas show an expansion trend in most scenarios, particularly under the SSP5-8.5 scenario, where highly suitable areas increase significantly while moderately suitable areas gradually shrink. To address future climate change, conservation strategies should focus on protecting highly suitable areas and strengthening the management of marginal habitats to enhance the adaptability and survival chances of *T. aeacus*.

## Simple Summary

This study was conducted to examine how the future distribution of *Troides aeacus*, a butterfly species, may be affected by climate change under different scenarios. It was found that temperature and precipitation are the main factors influencing its suitable habitat. By the 2050s and 2090s, both low and highly suitable areas are expected to expand, particularly under severe warming conditions (SSP5-8.5), while moderately suitable areas are likely to shrink. This shift could result in potential risks, such as habitat loss, increased competition, and local population declines. To ensure the species’ survival, conservation strategies should be focused on protecting optimal habitats and effectively managing marginal areas.

## 1. Introduction

*Troides aeacus* is the most northerly species in the Troides/Trogonoptera/Ornithoptera clade, known for its large size and golden–yellow hindwings. It is divided into five subspecies: the *Troides aeacus aeacus*, the *Troides aeacus formosanus*, the *Troides aeacus insularis*, the *Troides aeacus malaiianus*, and the *Troides aeacus szechwanus* [1]. *T. aeacus* is internationally recognized as one of the most ornamental butterfly species [2]. Its larvae feed on Aristolochiaceae plants, particularly those of the genus *Aristolochia*, such as *Aristolochia acuminata* and *Aristolochia foveolata*. Adults prefer to live in hot jungles, valleys, hills, and subtropical climates [3]. Due to differences in habitat and diet between larval and adult stages and sensitivity to environmental changes, *T. aeacus*’s survival is affected by numerous factors [4,5]. Consequently, it is listed as a protected species under the Convention on International Trade in Endangered Species of Wild Fauna and Flora (CITES) and as a second-class protected animal in the China National Key Protected Wildlife List [6]. Due to a decrease in habitat adaptability, the population of *T. aeacus* has been continuously declining [7]. Thus far, research on the potential suitable distribution range of *T. aeacus* has been relatively limited [8]. Therefore, conducting studies on the suitable distribution areas of *T. aeacus* in China is particularly important.

Climate change has the potential to affect species’ geographic distribution, abundance, distribution patterns, interspecies relationships, phenology, and photosynthesis, and it disrupts the balance of ecosystems [9]. Warmer temperatures can lead to increased metabolic rates, affecting the survival and reproduction of species. For some, this may mean population booms, while for others, it could signal a decline or even local extinction. The distribution patterns of species are further complicated by these changes, as habitats that were once suitable may no longer support the same diversity of life. For wildlife, climate change-induced habitat loss, food shortages, and invasive species pose significant survival challenges. Research indicates that the loss of various life forms has substantial impacts on the structure and function of ecosystems and affects ecosystem services [10]. This is particularly concerning for rare and endangered wildlife, as their lower abundance and often restricted and fragmented geographic ranges make them more vulnerable to environmental changes and extinction if not adequately protected [11,12].

The relationship between climate change and species distribution has long been a focal point of research. Predicting changes in suitable habitats for protected species under climate change is crucial for understanding species development patterns and establishing endangered species protection systems [13,14]. Therefore, studying the distribution dynamics of endangered species is particularly important for species assessment and conservation [15]. However, relying solely on field survey data often fails to capture the overall distribution trends of species comprehensively. By collecting known geographic distributions and corresponding environmental variables of species, suitable habitats can be predicted. Recently, Species Distribution Models (SDMs) have been widely used for predicting potential habitat distributions and have become an important tool for studying species suitability [16,17]. The most commonly used Species Distribution Models include Generalized Linear Models (GLM), Genetic Algorithm for Rule-set Prediction (GARP), Maximum Entropy (MaxEnt), HABITAT, and BIOCLIM. Each model has its advantages and limitations due to differences in their principles and algorithms [18,19]. When the relationship between species and environmental conditions is complex or the species’ distribution range is large, the Maximum Entropy model (MaxEnt) often provides better predictive accuracy compared to other models [20]. MaxEnt software (v3.4.4), which models species distribution based on recorded points, performs well even with small sample sizes and sparse distribution points and is widely recognized in the field for its short running time, simplicity of use, and minimal sample requirements [21,22,23].

Currently, research on the *T. aeacus* is still limited, with existing studies primarily focusing on its biological characteristics and habitat requirements. Research on the suitability assessment and protection of potential habitat distributions has not been given sufficient attention [24]. Therefore, this study uses the MaxEnt model to explore the relationship between the distribution of the *T. aeacus* and climatic conditions in China. It predicts the current and future potential distributions of the species and analyzes the centroid shift trend of its potential geographic distribution, providing important references and theoretical foundations for developing effective conservation measures.

## 2. Materials and Methods

### 2.1. Species Distribution Data

**Figure 1:**
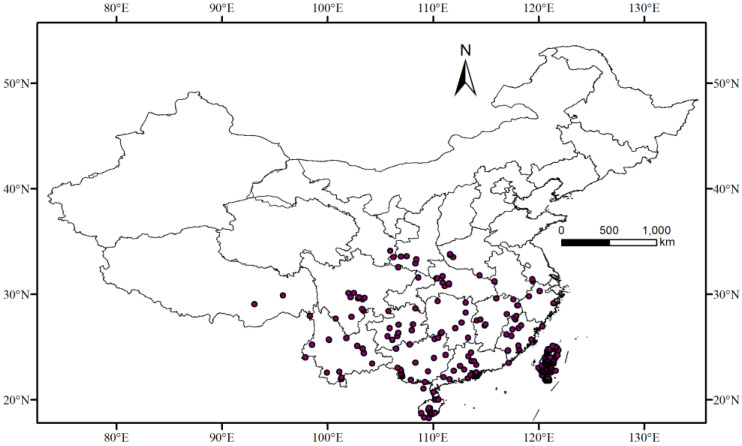
Distribution record of *T. aeacus* in China. (The red dots indicate the distribution points of *T. aeacus* in the current period).

All location data for this study are from China. The MaxEnt model simulation requires species distribution data and environmental data. Distribution data for the *T. aeacus* were obtained from the following: (1) specimens in research institution collections; (2) relevant literature records; and (3) the Global Biodiversity Information Facility (GBIF, http://www.gbif.org/) (accessed on 10 October 2024). Using Google Maps (http://ditu.google.cn) (accessed on 10 October 2024), these data were converted into geographic coordinates, resulting in a total of 490 sample points. To prevent model overfitting, if the distance between two distribution points was less than the environmental factor grid resolution (1 km), one of the points was removed [25]. Ultimately, 234 distribution records of the *T. aeacus* were selected. These distribution points were compiled in an Excel sheet with species name, longitude, and latitude in decimal format and saved as a “CSV” file for MaxEnt model calculations (Figure 1).

### 2.2. Environmental Variables and Data Processing

In this study, 22 environmental variables were selected, including 19 bioclimatic variables (bio1–bio19) and three terrain factors (Table 1). Environmental data were downloaded from WorldClim (https://www.worldclim.org/) (accessed on 10 October 2024), with “current period” data from 1970 to 2000 and future climate data for the 2050s (2041–2060) and 2090s (2081–2100) [26]. For future climates, this study chose SSP1-2.6, SSP2-4.5, and SSP5-8.5, representing low, medium, and high greenhouse gas emission scenarios, respectively, provided by the Beijing Climate Center’s new generation climate model BCC-CSM2MR [27]. Due to strong autocorrelation among environmental variables and the fact that not all variables are necessary for species distribution prediction [28,29], bioclimatic variables with high similarity to others or lower predictive capability were excluded to avoid overfitting and improve model accuracy [30]. Pearson correlation coefficients (R) were used to identify multicollinearity, and variables contributing significantly to model development were selected to minimize the impact of covariance on modeling and result interpretation [31,32]. When the absolute correlation coefficient between two ecological factors was ≥0.8, one representative variable was retained [33,34]. 

### 2.3. MaxEnt Model Construction and Result Evaluation

In this study, we used MaxEnt version 3.4.1 to model the current and future (2050s and 2090s) potential habitats of the *T. aeacus*. We imported the filtered distribution points into ArcGIS 10.8 and used the “Toolbox/Spatial Analyst Tools/Extraction/Sample” tool to sample and interpolate the distribution points across 22 environmental variables. The same method was applied to the initial MaxEnt prediction model for further sampling and interpolation of the distribution points. In SPSS, we conducted multicollinearity analysis using the Variance Inflation Factor (VIF) and Spearman correlation analysis on the interpolated data, initially selecting environmental factors with a VIF of less than 100 and a correlation of less than 0.8. VIF, also known as the reciprocal of tolerance, indicates multicollinearity: when VIF < 10, there is no multicollinearity among factors; when 10 < VIF < 100, moderate multicollinearity exists; and when VIF > 100, severe multicollinearity is present [35,36]. Environmental variables are crucial parameters in constructing niche models, as redundant variables can lead to overfitting, reducing model accuracy [37]. In this study, we used ENMTools.pl to analyze the correlation among the 22 environmental variables, defining variables with a Pearson correlation coefficient > |0.8| as highly correlated [38]. By comparing the contribution rates of variables in the initial model, we retained those with higher contributions and easier interpretability [39,40].

Based on statistical significance (partial receiver operating characteristic, ROC, with 500 iterations), predictive capability (omission rate, OR), and model complexity (AICc), we optimized the MaxEnt model parameters using the kuenm_ceval function [41]. According to the “OR_AICc” standard, a significant candidate model is defined as one with an omission rate below the threshold (e.g., ≤0.05, where applicable) and the lowest ΔAICc value (≤2), which is considered the final model with optimal parameters [42]. MaxEnt model parameters include feature combinations (FC), regularization multipliers (RM), and the maximum number of background points (BC), among others [43]. MaxEnt supports five feature types: linear (L), quadratic (Q), hinge (H), product (P), and threshold (T) [44]. By default, the RM value is set to 1, and the selection of feature combinations depends on the number of species occurrence points. Generally, the linear feature is always enabled; quadratic features are used when occurrence points exceed 10, hinge features are used when points exceed 15, and threshold and product features are applied when points exceed 80 [45]. However, studies have shown that the default MaxEnt settings are not always suitable for predicting all species distributions, potentially resulting in overfitting and difficulties in interpreting the predictions [46].

In this study, we used the ENMeval package in R 3.6.3 to optimize the MaxEnt model. Initially, we used a block partitioning method, dividing the distribution points of *T. aeacus* into four parts, with three parts used for training and one part for testing [47,48]. Subsequently, we utilized the kuenm package in R 3.6.3 to compare various combinations of the two key parameters (feature class and regularization multiplier) to identify the optimal combination [49]. The five MaxEnt features yielded 31 feature combinations, while the regularization multiplier was tested across four values ranging from 0.1 to 4 in 0.1 intervals. A total of 1240 candidate models were evaluated, including all combinations of 40 regularization multiplier settings, 31 feature classes, and a set of 11 environmental variables.

The receiver operating characteristic (ROC) curve is used to assess the accuracy of model predictions, with the Area Under the Curve (AUC) under the ROC curve serving as a performance indicator for MaxEnt predictions [50]. The AUC evaluation criteria are: 0.5 ≤ AUC < 0.6 indicates poor prediction; 0.6 ≤ AUC < 0.7 indicates fair prediction; 0.7 ≤ AUC < 0.8 indicates good prediction; 0.8 ≤ AUC < 0.9 indicates very good prediction; AUC > 0.9 indicates excellent prediction [51]. The closer the AUC value is to 1.0, the higher the accuracy of the model, allowing for the identification of the best predictive model.

### 2.4. Suitable Grade Zoning

Based on the actual situation of the *T. aeacus*, we used MaxEnt modeling and ArcGIS software to generate the probability distribution map of *T. aeacus* in China. To classify the levels of distribution values and their corresponding distribution ranges, the “Reclassify” function in ArcGIS was used according to the probability classification method recommended by the Intergovernmental Panel on Climate Change (IPCC) report [52]. Suitable habitats were categorized into four levels, with specific classification criteria as follows: 0–0.05 (unsuitable area); 0.05–0.33 (low-suitability area); 0.33–0.66 (moderate-suitability area); and 0.66–1 (high-suitability area) [53]. In the map, white, blue, orange, and red represent unsuitable areas, low-suitability areas, moderate-suitability areas, and high-suitability areas, respectively. Additionally, the SDMtoolbox in ArcGIS 10.8 (http://www.sdmtoolbox.org/) (accessed on 10 October 2024) was used to analyze the centroid positions and migration trends in *T. aeacus*’s potential distribution under different future climate scenarios [54].

## 3. Results

### 3.1. Model Performance and Key Environmental Variables

**Figure 2:**
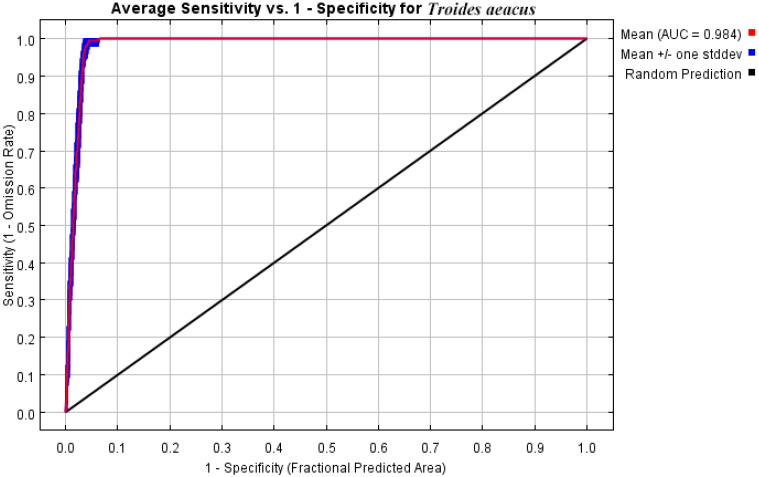
ROC curve of the potential distribution of *T. aeacus*.

By running the kuenm package in R 3.6.3, we identified only one model that met both the OR and AICc criteria. Consequently, for the MaxEnt model settings of *T. aeacus*, we selected the model M_0.1_F_pq_Set_1 (regularization multiplier = 0.1, feature combination = P and Q). Using 234 current distribution records and 9 environmental variables, we simulated the potential geographic distribution of *T. aeacus* in China with the MaxEnt software. The AUC value for the training data was 0.984 (Figure 2), representing an “excellent” level of performance. This indicates that the MaxEnt model’s predictions are accurate and reliable, demonstrating high predictive capability.

### 3.2. The Main Environmental Factors Influencing the Distribution of T. aeacus

**Figure 3:**
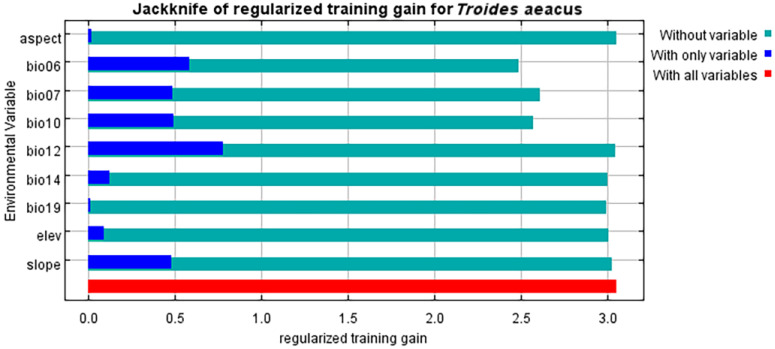
Jackknife test of the importance of environmental variables for the *T. aeacus*.

The MaxEnt model results identified the key environmental factors influencing the distribution range of *T. aeacus* and their respective contributions. The contribution rates of various environmental variables reflect their importance in determining species distribution. Among all the variables, the annual temperature range (bio7) plays a dominant role with a contribution rate of 26.50%, highlighting the significant influence of temperature variation on habitat selection. This is followed by annual precipitation (bio12), with a contribution rate of 20.10%. The minimum temperature of the coldest month (bio6) and slope contribute 15.00% and 10.00%, respectively, indicating that temperature extremes and terrain features also significantly impact habitat suitability. Additionally, the precipitation of the coldest quarter (bio19) contributes 10.00%, while the mean temperature of the warmest quarter (bio10) and precipitation of the driest month (bio14) contribute 7.90% and 6.70%, respectively, underscoring the role of seasonal climate in species adaptation. In contrast, altitude (elev) and aspect have lower contribution rates of 3.10% and 0.80%, respectively, suggesting a relatively minor influence on species distribution. Overall, temperature and precipitation emerge as the key determinants of species distribution, while terrain and other environmental variables have a comparatively secondary role (Table 2). The Jackknife test results indicated that when using single environmental variables, the five most influential variables were as follows: temperature annual range (bio5–bio6) (bio7), annual precipitation (bio12), min. temperature of the coldest month (bio6), slope (slope), and precipitation of the coldest quarter (bio19) (Figure 3).

### 3.3. Environmental Variables Influencing the Geographic Distribution of the T. aeacus

Based on the response curves of the probability distribution of the *T. aeacus* depicted by the MaxEnt model (Figure S1), the value ranges for variables affecting the future distribution of *T. aeacus* were determined. The results show that the range for temperature annual range (bio5–bio6) is 0.16 °C to 22.84 °C. Annual precipitation ranges from 2080.5 mm to 6324.6 mm. The range for the minimum temperature of the coldest month is 6.89 °C to 25.03 °C. Slope varies between 5.37° and 30.12°. The precipitation of the coldest quarter ranges from 81.83 mm to 2410.38 mm. Variations in these influencing factors have a significant impact on the presence probability of *T. aeacus*. If values exceed these ranges, the probability of the species’ distribution will gradually decrease.

### 3.4. Potential Distribution of the T. aeacus in the Current Period

**Figure 4:**
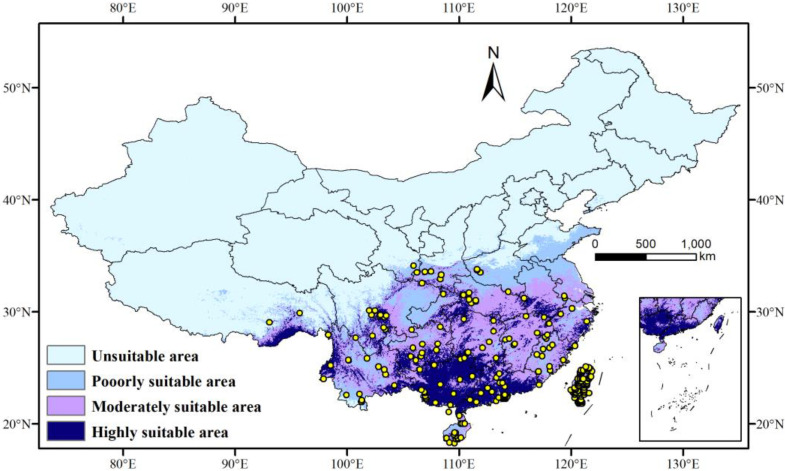
Distribution of suitable areas for the *T. aeacus* in China under current climatic conditions. (dark blue: high-suitability area; purple: medium-suitability area; blue: low-suitability area; and light blue: unsuitable area. The yellow solid dots represent the distribution points of *T. aeacus*).

Based on the MaxEnt model simulations using nine key environmental variables and distribution data of the *T. aeacus*, the potential distribution areas for the current period were classified into four suitability levels: high suitability, medium suitability, low suitability, and unsuitable. The results, which are illustrated in Figure 4, show a clear spatial gradient in the geographic distribution of suitable habitat for *T. aeacus*. The high-suitability areas are mainly concentrated in southern China and the southeastern coastal regions, including Guangdong, Guangxi, Hainan, Yunnan, Guizhou, and parts of the mid-to-lower Yangtze River basin, where environmental conditions are most favorable. Medium-suitability areas are widely distributed across the Yangtze River basin and its surrounding central and eastern regions, such as Hunan, Jiangxi, northern Zhejiang, and Jiangsu, where conditions are slightly less optimal but still supportive of the species’ growth. Low-suitability areas are scattered across the southern margins of North China, transition zones north of the Yangtze River, and the high-altitude edges of the southwestern region, where harsher climatic conditions limit the species’ wider distribution. Overall, the suitable habitat for *T. aeacus* displays a “southern concentration, northern dispersion” pattern, with suitability gradually decreasing as latitude increases.

The total area of suitable habitat for the *T. aeacus* in the contemporary period is 270.96 × 10^4^ km^2^ (Table 3). Among these, high-suitability areas, medium-suitability areas, and low-suitability areas cover 74.2 × 10^4^ km^2^, 113.03 × 10^4^ km^2^, and 83.73^4^ km^2^, respectively, representing 27.38%, 41.71%, and 30.90% of the total suitable habitat area.

### 3.5. Potential Future Distribution of the T. aeacus

**Figure 5:**
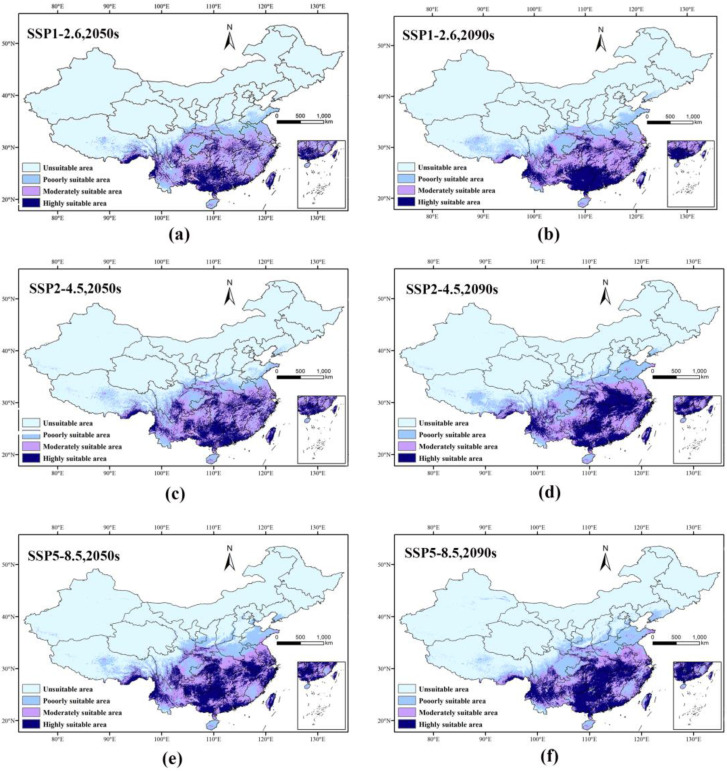
Comparison of potential distribution areas for *T. aeacus* between 2050 and 2090. (**a**) SSP1-2.6 (2050s); (**b**) SSP1-2.6 (2090s); (**c**) SSP2-4.5 (2050s); (**d**) SSP2-4.5 (2090s); (**e**) SSP5-8.5 (2090s); and (**f**) SSP5-8.5 (2090s).

Figure 5 illustrates the predicted distribution of suitable habitats for the *T. aeacus* under three climate change scenarios—SSP1-2.6, SSP2-4.5, and SSP5-8.5—for the 2050s (2041–2060) and 2090s (2081–2100). In addition, the areas of different suitability zone classes were also calculated (Table 3). In the 2050s, under different climate scenarios, the suitable habitat of *T. aeacus* shows a significant trend of change. The low-suitability area expands across all scenarios, with the most notable increase in the SSP5-8.5 scenario, where the area grows by 171,600 km^2^, representing a 20.49% increase compared to the current distribution. The SSP1-2.6 and SSP2-4.5 scenarios exhibit smaller expansions, with increases of 1400 km^2^ (0.17%) and 300 km^2^ (0.04%), respectively. The medium-suitability area shows minimal change in the 2050s, with decreases of 300 km^2^ (−0.03%) and 200 km^2^ (−0.02%) under SSP1-2.6 and SSP2-4.5 scenarios, respectively. However, it declines by 69,800 km^2^ (−6.18%) under the SSP5-8.5 scenario. In contrast, the high-suitability area expands overall, with increases of 2000 km^2^ (0.27%) and 487,900 km^2^ (65.77%) under SSP2-4.5 and SSP5-8.5, while remaining stable under SSP1-2.6.

In the 2090s, changes in the suitable area intensify across all scenarios. The low-suitability area expands significantly, with the largest increase under the SSP5-8.5 scenario, where it grows by 263,300 km^2^, representing a 31.43% increase from the current extent. SSP1-2.6 and SSP2-4.5 also show considerable expansions of 154,100 km^2^ (18.41%) and 196,700 km^2^ (23.49%), respectively. Meanwhile, the medium-suitability area decreases more sharply, with reductions of 59,200 km^2^ (−5.24%) and 76,200 km^2^ (−6.74%) under SSP1-2.6 and SSP2-4.5, respectively, and a substantial decline of 203,900 km^2^ (−18.04%) under SSP5-8.5. The high-suitability area experiences significant expansion in the 2090s, particularly under SSP5-8.5, increasing by 555,000 km^2^ (74.78%). It also grows under SSP2-4.5 and SSP1-2.6, with increases of 451,600 km^2^ (60.86%) and 142,800 km^2^ (19.25%), respectively.

### 3.6. Migration of the Centroid of Potential Distribution for the T. aeacus

**Figure 6:**
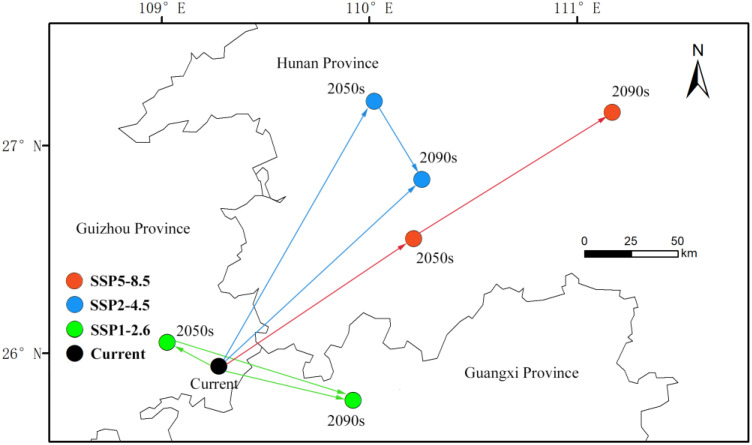
The change in the centroid of the potential distribution area of *T. aeacus* in China.( The black dots represent the center of mass of the current period, the green dots represent the movement of the center of mass of SSP1-2.6, the blue dots represent the movement of the center of mass of SSP2-4.5, and the red dots represent the movement of the center of mass of SSP5-8.5).

Under different climate scenarios, the centroid of the suitable habitat for *T. aeacus* shows significant spatial changes. Overall, as climate warming intensifies, the centroid tends to shift from northwest to southeast or northeast, but the specific trends vary across scenarios (Figure 6). In the SSP1-2.6 scenario, the centroid first shifts slightly northwest by 28.01 km from the present to the 2050s, with a direction of 297.3 degrees. However, from the 2050s to the 2090s, it shifts southeast by 94.99 km, with a direction of 108.92 degrees, indicating a transition from northwest to southeast due to future warming. In the SSP2-4.5 scenario, the centroid consistently moves northeast over all three periods: 115.89 km from the present to the 2050s, 116.58 km from the 2050s to the 2090s, and a total of 232.47 km from the present to the 2090s, with directions of 53.62, 54.27, and 53.74 degrees, respectively, suggesting a stable northeast shift under moderate warming conditions. In the SSP5-8.5 scenario, the centroid moves rapidly northeast by 160.04 km before the 2050s, with a direction of 27.52 degrees, representing the most pronounced early shift among all scenarios. However, from the 2050s to the 2090s, it shifts southeast by 47.5 km, with a direction of 151.58 degrees, indicating a temporal change in the expansion direction under extreme warming. Over the entire period from the present to the 2090s, the centroid moves 139.58 km northeast, with a direction of 43.98 degrees, demonstrating a long-term trend in expansion toward the northeast (Table 4).

The direction of centroid movement in the suitable habitat varies under different climate scenarios. In the SSP1-2.6 scenario, the centroid shifts northwest in the short term but transitions southeast in the long term. In the SSP2-4.5 scenario, the centroid exhibits a consistent shift toward the northeast. In the SSP5-8.5 scenario, the centroid rapidly expands to the northeast before the 2050s but gradually shifts southeast by the 2090s.

## 4. Discussion

This study, based on the MaxEnt model and ArcGIS geographic information technology, analyzes the current suitable distribution locations of the protected wildlife species in China, the *T. aeacus*, and predicts the future potential suitable areas while exploring the ecological characteristics of these regions. The model evaluation shows an AUC value of 0.962, indicating high accuracy. The results classify the *T. aeacus* into four different habitat suitability zones: high-suitability, moderate-suitability, low-suitability, and unsuitable zones. The projected climate change scenarios indicate that *T. aeacus*-suitable habitats will experience significant spatial shifts by 2050 and 2090. Climate change is expected to substantially expand the species’ highly suitable habitat areas, particularly under the SSP5-8.5 scenario, where the high-suitability range is anticipated to increase by 555,000 km^2^ by 2090—a 74.78% growth. This expansion of highly suitable areas suggests that *T. aeacus* may benefit from more optimal habitats under warming conditions, supporting population stability and growth. Furthermore, the dynamic nature of climate change and the increase in extreme weather events introduce uncertainties, potentially causing spatial and temporal fluctuations in habitat suitability. While the significant expansion of highly suitable areas may offer short-term advantages for population growth, the ecological stability of these expanded regions remains challenged by the impacts of ongoing climate change. Future research should incorporate these complex factors to better understand how shifts in habitat suitability may influence the long-term survival of *T. aeacus*. Although this study did not incorporate anthropogenic factors, human activities such as habitat loss, land-use changes, pollution, and resource competition may significantly impact the habitat suitability of *T. aeacus*, leading to habitat fragmentation, reduced ecological adaptability, and increased survival pressure. Therefore, future research should consider these human-induced disturbances to more comprehensively assess their potential effects on the distribution and population dynamics of *T. aeacus*.

Generally, the ecological niche of a species remains relatively stable over short historical periods, with minimal evolutionary changes [55]. Environmental variables affecting species distribution at varying spatial scales often show that, at larger scales, species interactions weaken and climate variables play a predominant role [56]. Research indicates that the choice of environmental variables can impact the predictions of ecological niche models [57]. Using the 22 bioclimatic factors from the WorldClim database, this study addressed the issue of unavoidable autocorrelation and multicollinearity among variables by performing correlation analysis and selection. Jackknife tests combined with Pearson correlation coefficients identified nine key environmental variables that limit the distribution of the *T. aeacus*: bio7, bio12, bio6, slope, bio19, bio10, bio14, elev, and aspect. The MaxEnt model was reconstructed to reduce redundant information and improve accuracy. Results showed that temperature and precipitation are significant factors influencing the distribution of *T. aeacus* [58]. Previous studies have shown that the *T. aeacus* prefers to live in hot jungles and valleys, being most commonly found during the rainy season. These areas have an annual temperature range of 16 °C–35 °C and an annual rainfall of 1500 mm to 2000 mm. The slightly increased annual precipitation observed in this study compared to past studies may be linked to global warming, which allows the atmosphere to hold more moisture, often resulting in increased precipitation.

As climate change and human activities impact the environment, biodiversity crises are occurring globally, with habitat fragmentation and loss being major drivers of biodiversity decline and species extinction [59]. Understanding the potential geographic distribution of threatened species and their current habitat suitability is crucial for effective conservation efforts. However, many species, especially endangered ones like the *T. aeacus*, have limited geographic distribution data. Based on the MaxEnt model predictions, the northward shift of the centroid suggests that the species’ suitable habitat may gradually expand to cooler northern regions in response to climate change or other environmental pressures, potentially as an adaptation to new ecological conditions or challenges brought about by changing climates.

## 5. Conclusions

Based on the MaxEnt model and species data distribution, the current and future suitable habitat distribution areas of the *T. aeacus* in China were determined. The results show that the min. temperature of the coldest month (bio6), temperature annual range (bio7), mean temperature of the warmest quarter (bio10), annual precipitation (bio12), precipitation of the coldest quarter (bio19), and slope affect its distribution. The main environmental factors of the pattern. Climate change will significantly affect its distribution pattern. Temperature and precipitation are the main factors affecting its distribution pattern. Habitat expansion and contraction are closely related to these indicators. Under the current climate conditions, the *T. aeacus* is mainly distributed in the area south of the Huaihe River in the Qinling Mountains. The high-suitability areas are mainly distributed in Guangxi, Guangdong, Hong Kong, and Taiwan, with the highly suitable area reaching 74.2 × 104 square kilometers. Under future climate conditions, the low- and high-suitability areas are expected to expand significantly, especially under the SSP5-8.5 scenario in the 2090s; they increased by 26.33% and 55.5%, respectively. Therefore, relevant managers can use these habitat suitability maps to identify high-risk areas, thereby prioritizing conservation actions in these areas and facilitating the smooth implementation of habitat assessment and protection work.

## Figures and Tables

**Table 1:** Environmental variables of potential geographical distribution of *T. aeacus*.

Symbol	Environmental Variable	Unit
Annual mean temperature	bio1	°C
Mean diurnal range (monthly mean) (max. temp–min. temp)	bio2	°C
Isothermality (bio2/bio7) (×100)	bio3	°C
Temperature seasonality (standard deviation × 100)	bio4	°C
Max. temperature of the Warmest Month	bio5	°C
Min. temperature of the Coldest Month	bio6	°C
Temperature annual range (bio5–bio6)	bio7	°C
Mean temperature of the Wettest Quarter	bio8	°C
Mean temperature of the Driest Quarter	bio9	°C
Mean temperature of the Warmest Quarter	bio10	°C
Mean temperature of the Coldest Quarter	bio11	°C
Annual precipitation	bio12	mm
Precipitation of the Wettest Month	bio13	mm
Precipitation of the Driest Month	bio14	mm
Precipitation seasonality (coefficient of variation)	bio15	%
Precipitation of the Wettest Quarter	bio16	mm
Precipitation of the Driest Quarter	bio17	mm
Precipitation of the Warmest Quarter	bio18	mm
Precipitation of the Coldest Quarter	bio19	mm
Altitude	elev	m
Slope	slope	degree
Aspect	aspect	degree

**Table 2:** Permutation importance of model variables.

Code	Environmental Variables	Contribution Rate
bio7	Temperature annual range (bio5–bio6)	26.50%
bio12	Annual precipitation	20.10%
bio6	Min. temperature of the Coldest Month	15.00%
slope	Slope	10.00%
bio19	Precipitation of the Coldest Quarter	10.00%
bio10	Mean temperature of the Warmest Quarter	7.90%
bio14	Precipitation of the Driest Month	6.70%
elev	Altitude	3.10%
aspect	Aspect	0.80%

**Table 3:** Comparison of suitable habitat area for *T. aeacus* under current and future climate conditions.

		Predicted Area (10^4^ km^2^)			Comparison with Current Distribution (%)		
Period	Scenarios	Low-Suitability	Medium-Suitability	High-Suitability	Low-Suitability	Medium-Suitability	High-Suitability
Current	-	83.73	113.03	74.2	-	-	-
2050s	SSP1-2.6	95.53	109.46	74.22	0.14	−0.03	0
	SSP2-4.5	86.53	110.43	89.41	0.03	−0.02	0.2
	SSP5-8.5	98.1	105.14	110.4	17.16	−6.98	48.79
2090s	SSP1-2.6	96.63	107.41	84.82	15.41	−1.87	14.28
	SSP2-4.5	100.2	104.42	107.71	19.67	−7.62	45.16
	SSP5-8.5	105.78	89.98	115.38	26.33	−20.39	55.5

**Table 4:** Trajectory of centroid shift in the suitable habitat of *T. aeacus* under climate change scenarios.

Scene	Period	Angle (°)	Direction	Displacement (km)
SSP1-2.6	Current to 2050s	297.3	northwest	28.01
	2050s to 2090s	108.92	southeast	94.99
	Current to 2090s	105.6	southeast	67.39
SSP2-4.5	Current to 2050s	53.62	northeast	115.89
	2050s to 2090s	54.27	northeast	116.58
	Current to 2090s	53.74	northeast	232.47
SSP5-8.5	Current to 2050s	27.52	northeast	160.04
	2050s to 2090s	151.58	southeast	47.5
	Current to 2090s	43.98	northeast	139.58

## Data Availability

The data supporting the results are available in a public repository at: GBIF.org (12 April 2024), GBIF Occurrence Download https://doi.org/10.15468/dl.4gyzgp; *T. aeacus* occurrence data: 10.6084/m9.figshare.27309102. Inquiries regarding code availability and further inquiries can be directed to the corresponding author.

## Supplementary Materials

The following supporting information can be downloaded at: https://www.mdpi.com/article/10.3390/insects15110901/s1. Figure S1: Response Curves of the presence probability of the *T. aeacus* to key environmental variables.
