# Supplementary material for: Predicted Spatial Patterns of Suitable Habitats for Troides aeacus Under Different Climate Scenarios
Source: Insects. 2024 Nov 18;15(11):901. doi: 10.3390/insects15110901 (PMC11594763; doi:10.3390/insects15110901)
Supplement: Supplementary file 1 [file insects-15-00901-s001.zip › Supplementary File.pdf]

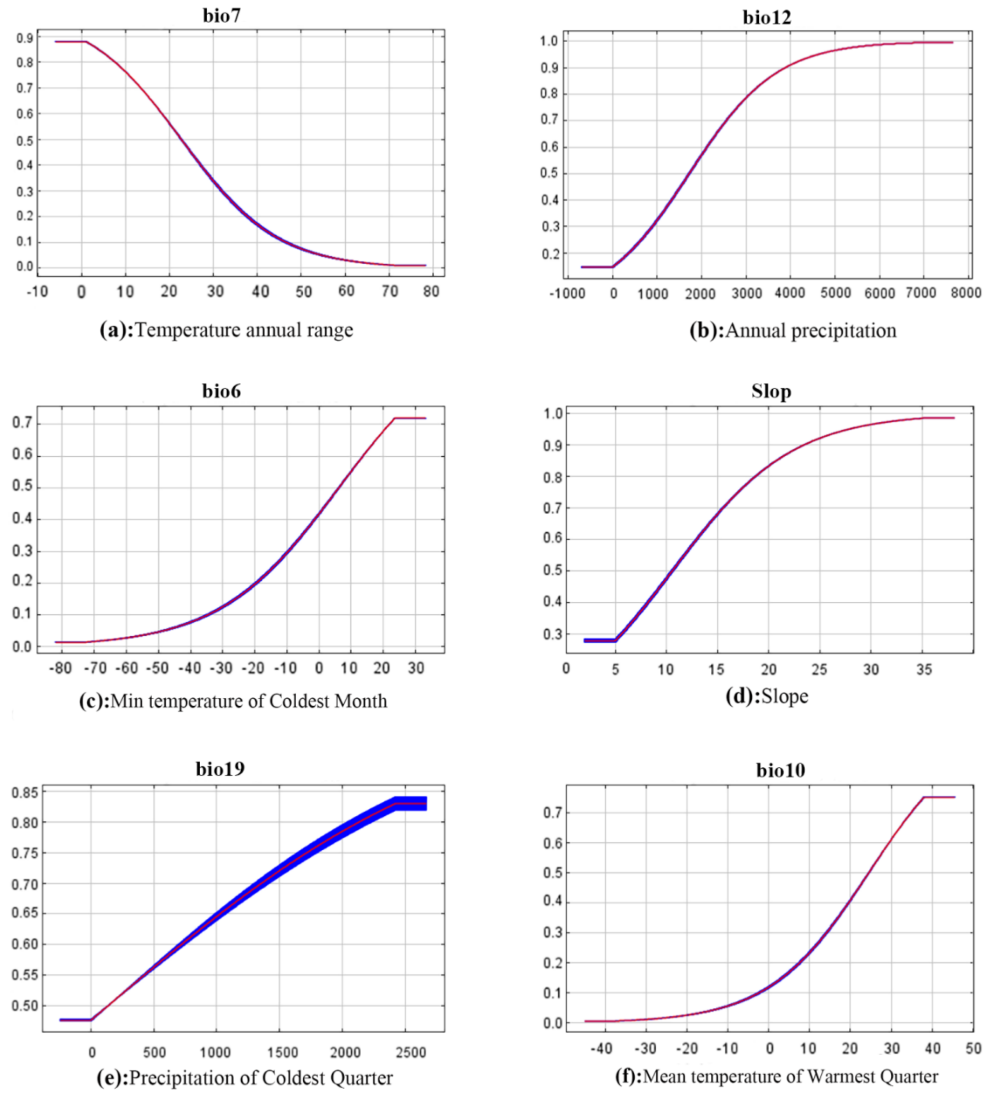

Figure S1. Response Curves of the presence probability of the *T. aeacus* to key environmental variables.
